# Optofluidic Force Induction: A Workbench for Nanoparticle Characterization and Material Analytics

**DOI:** 10.1021/acs.nanolett.5c01126

**Published:** 2025-05-21

**Authors:** Marko Šimić, Christian Neuper, Raphael Hauer, Karin Grießmair, Christian Hill, Ulrich Hohenester

**Affiliations:** † Institute of Physics, 27267 University of Graz, Universitätsplatz 5, 8010 Graz, Austria; ‡ Christian Doppler Laboratory for Structured Matter Based Sensing, Institute of Physics, Universitätsplatz 5, 8010 Graz, Austria; ¶ Brave Analytics GmbH, 8010 Graz, Austria; § Graz Centre for Electron Microscopy, Steyrergasse 17, 8010 Graz, Austria; ∥ Institute of Biomedical Imaging, Graz University of Technology, Stremayrgasse 16/III, 8010 Graz, Austria; ⊥ Gottfried Schatz Research Center, Division of Biophysics, Medical University of Graz, Neue Stiftingtalstraße 2, 8010 Graz, Austria

**Keywords:** Optofluidic force induction, Nanoparticle characterization, Raman, Rayleigh Scattering

## Abstract

Nanoparticle characterization in dispersion lies at the heart of modern research and industry, which is transitioning from batch-wise to continuous production. In many cases, manufacturers of nanoparticle-based products must comply with prescribed regulations and rely on precise knowledge and control of critical process parameters that directly affect the quality of a final product. In this Mini Review, we present Optofluidic Force Induction (OF2i) as a workbench for real-time nanoparticle characterization with single-particle sensitivity and high throughput. We discuss its underlying physical principles and demonstrate its capability for online process analytics and correlative particle analysis based on industrially relevant and complex samples. We elaborate on recent achievements and ongoing developments and discuss challenges and possible directions for future research. Our results prove that the correlative OF2i approach paves the way for a broad range of applications and opens up new avenues in both industry and research.

## Graphical abstract

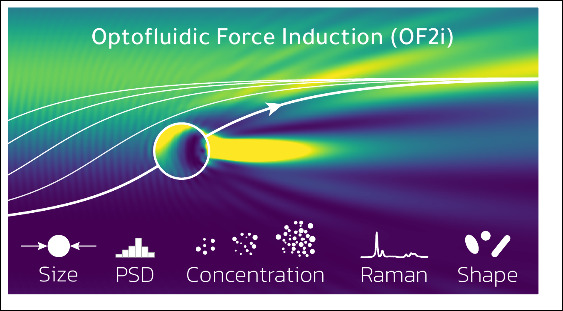

Process analytical technologies (PATs) remain an integral part of the regulatory compliance and quality assessment of nanoparticle-based products across a broad spectrum of industries and scientific research, reaching from pharmaceutics and nanomedicine to environmental sciences and semiconductor industries.^1^ In many cases, manufacturers must comply with prescribed regulations and rely on precise knowledge and control of critical quality attributes (CQA) throughout a production process to achieve the desired quality and performance of a final product, as emphasized in various guidelines issued by the European Medicines Agency.^2,3^ Driven by regulatory bodies and a transition from batch-wise to continuous production,^1,4^ the requirements on PATs drastically increased over the past years.

Central to these developments is the strong need for real-time nanoparticle characterization technologies, capable of providing information about CQAs such as particle size, particle size distribution (PSD), concentration, material analytics, or even basic shape information. While conventional techniques have proven to be reliable offline characterization methods in the past, they face challenges in providing accurate and fast process feedback to comply with regulations and *Quality by Design* approaches.^5^ This particularly applies to the characterization of nanoscaled materials^1^ and the detection of ultralow concentrated particles (e.g., USP 729 ^6^ and USP 787 ^7^ for injectables), which play a decisive role in the analysis of water for injectables,^8^ lipid emulsions,^9^ impurities in drinking water,^10,11^ and contaminants in environmental analytics.^12,13^

To address these demands, in the past years optofluidic force induction (OF2i)^14–17^ has been developed as a platform technology to deliver detailed information about nanoparticle characteristics and CQAs with single-particle accuracy and high throughput over a broad size range, even at ultralow concentrations. A combination of optical and fluidic forces allows OF2i to be operated in an online manner, where a small portion of the sample flow is extracted continuously via a bypass system, analyzed, and either returned to the process stream or redirected to waste. The real-time capabilities of the system allow measuring changes in process dynamics and CQAs on time scales ranging from a few seconds up to several hours.^16^ Thus, OF2i enables fast and robust particle analysis without process interruption, while its modular setup allows for complementary particle analytics.

In this mini review, we start with a brief overview of the most common and well-established optical particle characterization techniques and discuss their underlying basic physical principles. This sets the stage for the main part of this review, where we focus on the more specialized OF2i nanoparticle characterization technique, operating online and in real time. OF2i uses light not only for observation purposes but also as a tool to obtain nanoparticle properties from optical forces using the optical tweezer principle. We discuss experimental and theoretical details and explore OF2i as a workbench, showcasing its various operation modes, which have been developed by the authors over the past years, and their combinations for correlative measurements on the example of industrially relevant samples. Prospects for ongoing developments, challenges, and an outlook on future directions conclude this review. The Supporting Information provides video material for the different OF2i operation modes.

## Optical Nanoparticle Characterization Schemes

In this section, we give a brief overview of optical characterization schemes for nanoparticles immersed in fluids. Our main motivation is to provide an overarching picture of the physical processes involved, rather than to present an exhaustive presentation of the many different implementations available. Common to all schemes is the interaction of a well-defined incoming light field, typically produced by a laser, with the nanoparticles to be analyzed and the observation of the scattered fields in order to infer specific properties such as the size, shape, or PSD. Here we adopt a generic description in terms of multipole coefficients *q*_inc_ and *a*_sca_ for the incoming and scattered fields,^18^ respectively, which are connected in linear response through the transition or T-matrix^19,20^

$$a_{\mathrm{sca}}=Tq_{\mathrm{inc}}$$ 1

The T-matrix depends on the size, shape, and material properties of the nanoparticle. Thus, by measuring the angular distribution of the scattered light (*a*_sca_) for a well-defined incoming field (*q*_inc_), one can infer information about the particle. This is utilized in static light scattering (SLS) and laser diffraction spectroscopy (LDS) by measuring the light scattered into different angular directions and comparing the intensities with theoretical predictions based on Mie or Fraunhofer theory for spherical particles,^21^ in order to obtain the size distribution of particles immersed in a fluid, as schematically shown in Figure 1(a,e,f).

**Figure 1:**
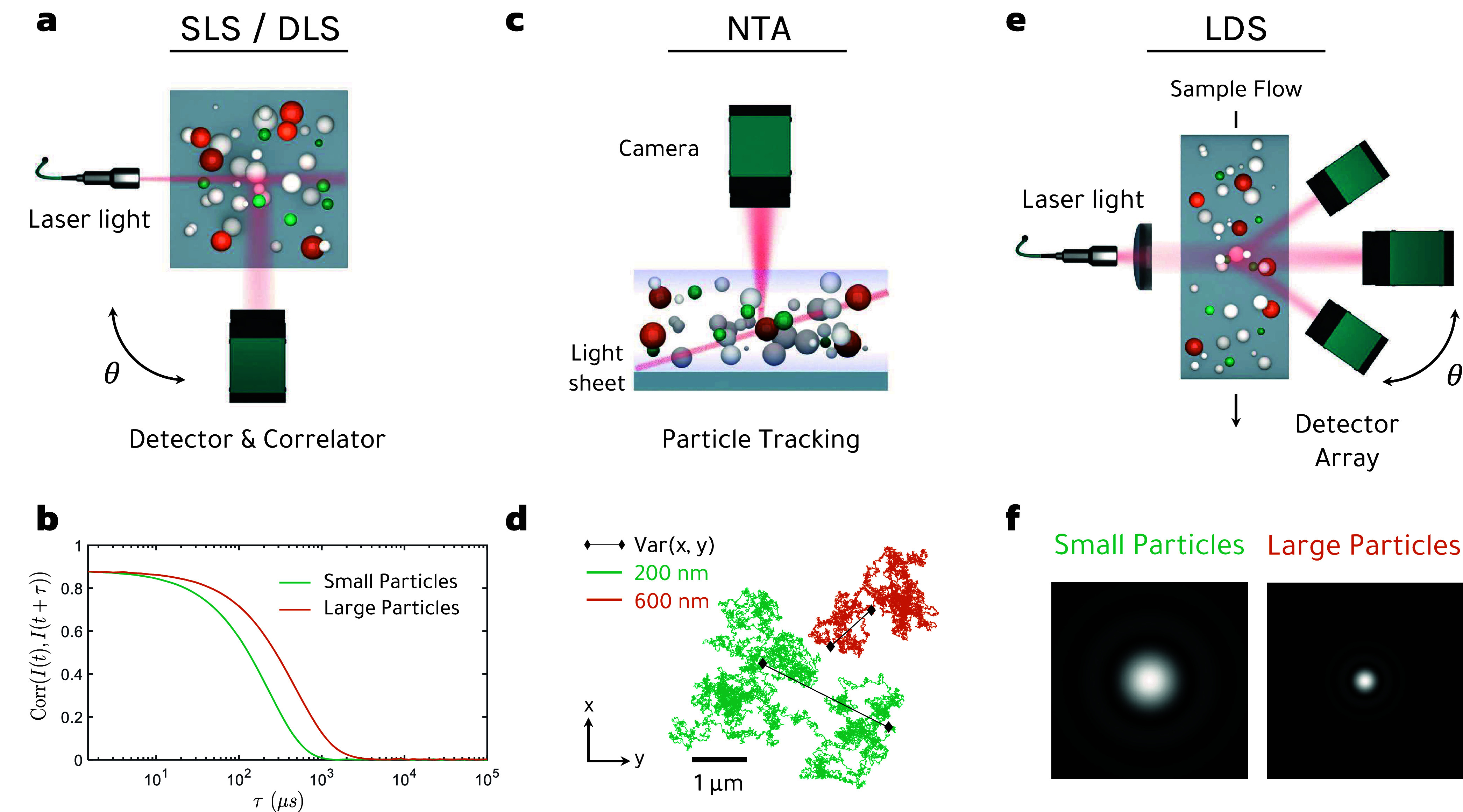
Overview of optical nanoparticle characterization techniques based on Brownian motion and light scattering. (a) Schematics of SLS and DLS, where one observes the scattered light of an ensemble of nanoparticles undergoing Brownian motion. From the angular intensity distribution (SLS) or the autocorrelation of intensity fluctuations over time, in DLS (b) one can extract information about particle size of the whole ensemble. (c) In NTA, scattered light of individual particles is tracked over time *t*, as shown on the example of a (d) 200 and 600 nm sized particle, respectively. The variance, or the displacement of a particle over time, provides a direct relationship to particle size; see main text for details. (e) In LDS, the predominantly forward-scattered light pattern of a particle ensemble is analyzed and compared with Mie’s theory or Fraunhofer diffraction, as shown on the example of two size classes in (f).

In contrast to SLS and LDS, in dynamic light scattering (DLS) information about the sizes of the particles to be analyzed is obtained from their dynamic time evolution due to Brownian motion. Newton’s equations of motion for a nanoparticle in a fluid are governed by

$$m\mathrm{v̇}=D\cdot v+F_{\mathrm{stoch}}\approx 0$$ 2

Here, *m* is the mass of the particle, which might include the added mass due to the fluid,^22^ ***v*** is the velocity of the particle, and **D** is the Stokes drag tensor. For a spherical particle, it has the usual form **D** = – 6*πηR* **I**, where η is the fluid viscosity, *R* the Stokes or hydrodynamic radius, which might account for additional solvent effects, and **I** the unit tensor. We assume that the momentum relaxation time is so short that we can approximately set ***v̇*** ≈ 0,^22^ which corresponds to a particle flow in the small Reynolds number regime.^23^ The stochastic force ***F***_stoch_ is needed to counterbalance the Stokes drag^24^ and leads to a random movement known as Brownian motion. The variance of nanoparticle trajectories starting from the same initial position increases over time according to var(x)=2Dt . The fluctuation–dissipation theorem establishes a relation between the diffusion coefficient D and the Stokes drag through $D=k_{B}T/\left(6\pi \eta R\right)$ . In DLS, one then measures the temporal evolution of light scattered by an ensemble of nanoparticles to be analyzed. Due to interference, the scattered light undergoes fluctuations, and due to Brownian motion, the fluctuations decay over time, as shown in Figure 1(b). Thus, from the intensity autocorrelation, information about the diffusion coefficient and size distribution of the particle ensemble can be obtained.^25^ Building on the concept of DLS, the recently introduced spatially resolved dynamic light scattering SR-DLS enables the continuous inline monitoring of concentrated nanodispersions, providing real-time information about particle size and PSD.^26^ This technique uses Fourier-domain low-coherence interferometry to obtain spatially resolved DLS data, taking into account both multiscattering events as well as sample flow.

Another prominent Brownian motion-based technique is provided by nanoparticle tracking analysis (NTA),^27–29^ where one tracks the positions of individual nanoparticles with a camera over some time period and then obtains from var­(*x*) an estimate for the diffusion coefficient and particle size, see Figure 1(c,d). Brownian motion can also be measured in interferometry scattering (iSCAT), a technique that was originally designed for the label-free detection of macromolecules, proteins, and other nanoparticles.^30,31^ Here the light scattered by the nanoparticles is brought to interference with a reference field, typically the part of the incoming fields that is reflected at a coverslip, which leads to a significantly enhanced signal-to-noise ratio. From the observation of the signal strength and the Brownian motion, it is possible to extract both the Stokes radius and the refractive index of single particles, a technique that has been termed iNTA.^32^

Although strictly speaking not a particle characterization scheme, optical tweezers can be used to trap and manipulate single nanoparticles. This technique was pioneered by Arthur Ashkin,^33^ who received in 2018 the Nobel Prize in Physics for his work on “optical tweezers and their application to biological systems”. Optical tweezers have become an indispensable tool in various fields of research, ranging from physics over biochemistry to (nano)­medicine.^34–39^ A tightly focused laser beam becomes partially scattered by the nanoparticle, and the momentum transferred from light to matter exerts forces in the pico-Newton range and allows trapping sufficiently small particles. Theoretically, the optical force ***F***_opt_ acting on the particle can be obtained from the solution of eq 1 by computing the flow of Maxwell’s stress tensor through a boundary enclosing the particle.^36,40^ Correspondingly, Newton’s equation of motion governing the dynamics of trapped particles is obtained from eq 2 by accounting for both optical and fluidic forces through

$$m\mathrm{v̇}=F_{\mathrm{opt}}\left(a_{\mathrm{sca}},q_{\mathrm{inc}}\right)+D\cdot v+F_{\mathrm{stoch}}\approx 0$$ 3

Conveniently, the optical force can be split into a scattering part, which pushes the particle in the laser propagation direction, and a gradient part, which pulls the particle into regions of high laser intensities.^36,40^ By modifying the incoming laser fields, for instance, with spatial light modulators or diffractive optical elements to generate structured light,^41^ one has an active control for manipulating single or multiple particles, which could eventually be exploited for nanoparticle characterization.^42,43^

## Optofluidic Force Induction

OF2i is a nanoparticle characterization scheme building on the optical-tweezer principle and combines single-particle sensitivity with high throughput and the capability of online measurements.^15,16,44^ This is achieved by pumping the nanoparticles to be analyzed through a microfluidic flow cell and sending a weakly focused laser beam in the flow direction (+ ***ẑ***), as schematically shown in Figure 2(a). By analyzing the Rayleigh scattering signal in the side image, one can infer information about particle size, size distribution, number, concentration, and even basic shape information.^15,44^ In the dynamic equation for the nanoparticles, we additionally have to account for the fluid velocity ***v***_fluid_ through

$$m\mathrm{v̇}=F_{\mathrm{opt}}\left(a_{\mathrm{sca}},q_{\mathrm{inc}}\right)+D\cdot \left(v-v_{\mathrm{fluid}}\right)+F_{\mathrm{stoch}}\approx 0$$ 4

The effect of the laser is 3-fold. First, particles in the vicinity of the focused laser scatter light and can be observed in an imaging system, see the *Side Image* in Figure 2. Second, through the gradient forces, particles can become trapped in the radial directions in regions of high field intensity, see panel (b), similar to optical tweezers. Third, the scattering forces in the laser propagation direction push the particles and lead to velocity enhancements that can be measured. When neglecting in eq 4 the stochastic forces and using for the Stokes drag the expression for a sphere, we can relate the particle velocity to the optical force through

$$v\left(r\right)=v_{\mathrm{fluid}}+\frac{F_{\mathrm{opt}}\left(r\right)}{6\pi \eta R}$$ 5

where we have indicated that, because of the spatially varying laser intensity profile, the optical force and particle velocity depend on the position ***r*** inside the flow cell. This expression lies at the heart of OF2i and allows us to relate the velocity of the particle to the diameter of the particle, as will be discussed in more detail below.

**Figure 2:**
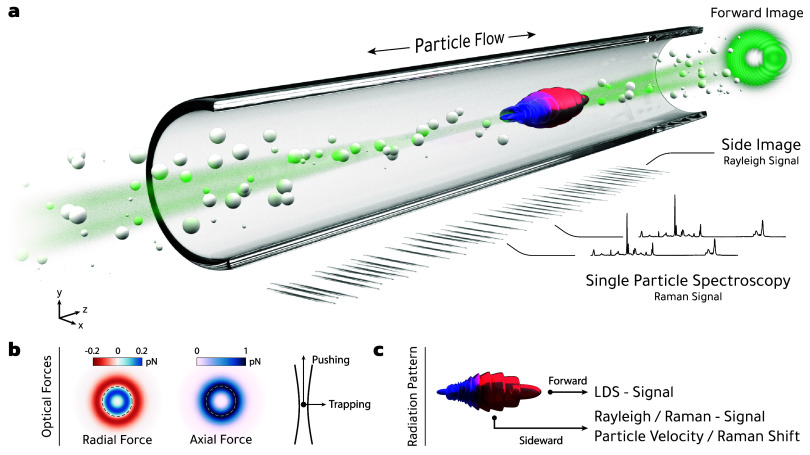
Working principle of the OF2i scheme. (a) Nanoparticles suspended in a liquid are continuously pumped through a microfluidic channel alongside a weakly focused vortex beam. The purpose of the laser beam is 3-fold: First, through optical forces in radial direction (b, left), nanoparticles sufficiently close to the intensity maxima become trapped in transverse direction (dashed line). Second, the optical forces in the laser propagation direction (b, right) push the particles and lead to velocity changes depending on size, shape, and material properties. Third, the light scattered off the particles is detected in the *Side Image*, which allows monitoring of the velocity changes through the Rayleigh scattering signal as well as analysis of Raman scattering. (c) Radiation pattern of a single particle, where the sideward-scattered light is mainly composed of the SLS- and Raman signals. Large particles additionally scatter light in the forward direction, resulting in a LDS-signal or causing obscuration, which is recorded through the *Forward Image*.

The OF2i experimental setup consists of a two-dimensional optical trap in a microfluidic flow channel of cylindrical symmetry, which is created by a 532 nm CW DPSS laser (Laser Quantum, GEM532). Beam alignment and preparation are achieved with two mirrors and a 5x beam expander. An azimuthally polarized vortex beam is generated using a zero-order vortex half-wave retarder (*q* = 1/2), resembling a vortex pattern of topological charge *m* = 1. A converging lens focuses the collimated beam into the measurement cell. A sample stream can be selected using a multiport valve, which is connected to the measurement cell and can be optionally fed through our in-house developed dilution system. A schematic of the OF2i setup is provided in Figure 7 of ref 15. The sideward-scattered light is captured by an ultramicroscope setup and redirected onto a camera that provides the raw video signal (see Side Image in Figure 2). In a second imaging path, the sideward-scattered light is spatially dispersed and focused onto a camera using two cylindrical lenses and a custom-built Raman spectrometer providing the raw Raman signal (see panel (a) of Figure 4). For light obscuration, we use a light-emitting diode as an excitation source and a camera to record forward scattered or obscured light. Control of laser power, flow direction, and valve position as well as real-time visualization of monitoring data is provided by Brave Analytics proprietary software suite HANS.

The top panel of Figure 3(a) shows one side image extracted from a recorded movie for a sample where standardized polystyrene (PS) nanospheres with different diameters are immersed in water and pumped through the microfluidic capillary, see also the video in the Supporting Information. Because of the cylindrical shape of the capillary, the light scattered by the spheres is imaged as vertical lines rather than points.^45^ In the following, we integrate the side image along the vertical direction and obtain a one-dimensional array that encodes the total scattered intensity at a given time and propagation distance along the observation window. The stacking of several arrays at different times forms the waterfall diagram shown in the lower part of the panel and reveals the trajectories of particles as they are transported from left to right. The larger particles, which appear brighter in the figure, move faster in the focus region (dashed line) due to axial forces, see eq 5. The vortex beam used in our experiments allows particles to overtake each other (as indicated by the arrow marking crossing trajectories), enabling parallel processing of both monodisperse as well as highly polydisperse and multimodal samples. In a next step, we determine from the slope of each trajectory the particle velocities along the flow cell to obtain the histogram shown in panel (b). Here, we can already identify five different particle populations with maximum velocity separation in the focus region of the vortex beam.

**Figure 3:**
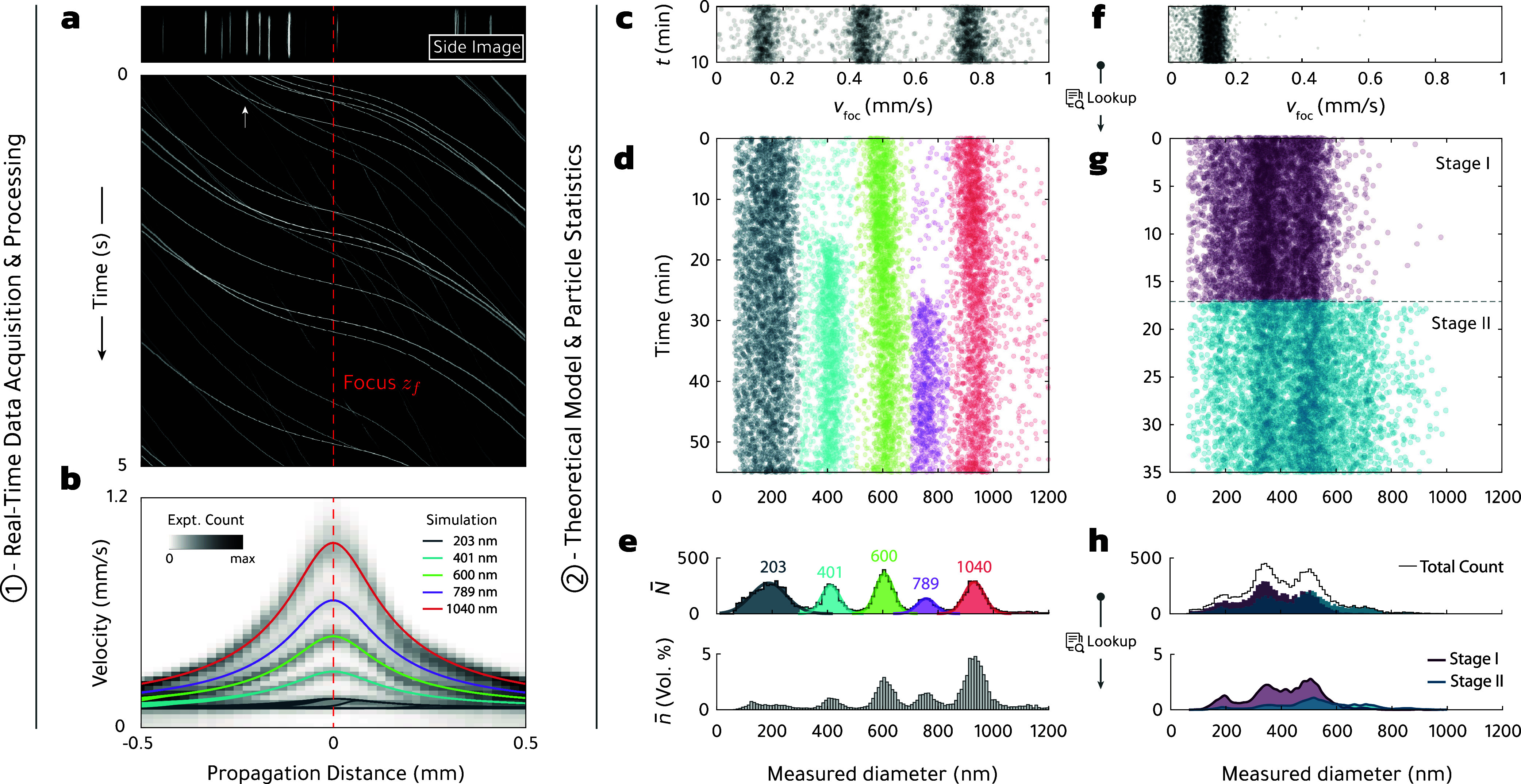
Data processing methods (1), modeling and particle statistics, and OF2i measurement results (2). (a) Light scattered off particles is recorded in the *Side Image* over time and converted into a waterfall diagram (partially shown), encoding the scattered light intensity of each particle as a function of propagation distance and time, resulting in single-particle trajectories. (b) Particle velocity histogram, obtained by measuring the slope of each trajectory as a function of propagation distance. Solid lines represent simulated particle trajectories of various sizes.^44^ (c) Time series of particle velocities extracted from (b) in the focal region of the vortex beam (dashed line) within the first 10 min of measurement. These act as an input for a lookup table, which relates theoretical velocities to particle sizes and these, in turn, to the corresponding active volume. (d) Scatter diagram of particle sizes, as obtained by continuous monitoring of a mix of polystyrene particles.^16^ (e) Particle count *N̅* and resulting PSD *n̅* (volume distribution). (f–h) Continuous monitoring of high-pressure homogenization states of an oil-in-water emulsion.^16^ Figure adapted with permission from refs 15 and 16 . Copyright American Physical Society and Springer Nature.

To corroborate our experimental results, we have developed a simulation approach^44,46^ that is closely related to that of optical tweezers.^36,47^ It starts by specifying the incoming electromagnetic fields of the focused laser beam, which are used to compute for a given particle position ***r*** the beam-shape coefficients *q*_inc_(***r***). In a second step, we solve the scattering problem of eq 1 for a particle with a specified refractive index and diameter, and obtain the scattered fields characterized by *a*_sca_. From the knowledge of *q*_inc_ and *a*_sca_, we get in a third step the optical force ***F***_opt_(***r***) and finally determine the particle trajectories from the solutions of eq 4, considering or neglecting Brownian motion. If needed, the scattered electromagnetic fields *a*_sca_ can be used to simulate the OF2i side image.^45^ The solid lines in Figure 3 show the simulated velocities for PS spheres of the same nominal diameters as used in the experiment, which agree well with the experimental data.

To relate the measured velocities in the focus plane *v*_foc_ to particle diameters, we start by performing simulations for different sphere diameters *d* while keeping all other simulation parameters, such as refractive index or laser beam coefficients, fixed. We will discuss below how additional information about the particles to be analyzed can be obtained from experiment. From the simulations we obtain the particle velocity in the focus region *v*_sim_(*d*) as a function of particle diameter. For sufficiently small particles with low refractive index contrast, Mie resonances play no significant role,^15,44^ and the velocity relation can be inverted in order to obtain the particle size from the particle velocity through *d* = *d*(*v*_sim_). In analyzing the experimental OF2i data, we store the relation as a lookup table and finally relate the measured velocities to diameters using *d*_meas_ = *d*(*v*_foc_).

Figure 3(c) shows the measured particle velocities of PS spheres over the first 10 min of a continuous monitoring process.^16^ Through the lookup table, we can translate the velocities to diameters, which are reported in panel (d). One observes that initially only spheres with diameters of 200, 600, and 1000 nm are present, while at later times we also mix PS spheres with diameters of 400 and 800 nm into the fluid that is pumped through the microfluidic channel. Panels (f,g) show corresponding data for an oil-in-water emulsion,^16^ where the target of the monitoring process is to discriminate between two stages in the production process.

In the top of Figures 3(e,h), we report the histograms for the measured particle counts using a mesh with a bin width of Δ*d*. When translating the measured particle count *N*(*d*, Δ*d*) to a PSD, we have to account for the fact that due to the size-dependent optical trapping efficiencies in the radial direction, larger particles are observed more frequently than smaller ones. To this end, we have introduced the concept of an active volume^15,16,44^

$$V_{\mathrm{active}}\left(d\right)=\left(\pi r_{\mathrm{cut}}^{2}\left(d\right)\right)v_{\mathrm{fluid}}t_{\mathrm{meas}}$$ 6

which is the volume sampled during the measurement time *t*_meas_. *r*_cut_(*d*) is a size-dependent cutoff radius that is determined from our simulations and gives the maximal radial distance at which particles of diameter *d* propagating along the fluid channel become trapped by the focused laser beam. After trapping, the particles are transported along the intensity maxima and are measured later in OF2i in the focus region. The PSD *n*(*d*) is obtained by dividing the particle count by the active volume,

$$\mathrm{n̅}\left(d\right)\Delta d=\frac{\mathrm{N̅}\left(d,\Delta d\right)}{V_{\mathrm{active}}\left(d\right)}$$ 7

Because each particle is counted individually as it flows through the measurement cell, OF2i can be categorized as an online counting method, and the resulting PSD is described on a number basis. Through eq 7, we are able to infer real-time information about (weighted) mean diameters, D-values, and trends, and can easily convert to other representation bases, such as volume or intensity weighted ones.^16^ The bottom panels of Figure 3(e,h) shows the volume-weighted PSD. Depending on the sampling time *t*_meas_, *n̅* can be represented as a single or rolling statistic over the entire time series.

## The OF2i Workbench

OF2i, as discussed in the previous section, provides information about particle properties from the observation of how they react to the combined effect of optical and fluidic forces. However, OF2i can also be seen in a broader context as a workbench for a variety of operation modes that combine light scattering with optical and fluidic forces. In the following, we discuss several modes that have been already implemented, are currently explored, or are planned for future investigations, see also Table 1 for an overview. It is important to note that the operation modes are not mutually exclusive but can complement each other.

**Table 1:** List of Different OF2i Operation Modes^a^

OF2i operation mode	optical forces	optical scattering	complementary operation	size range
(F) Flow	yes, velocity increase	tracking via side camera	C, R, M, line shape	>100 nm
(C) Correlative	yes, velocity increase	tracking via side camera	F, T, R, B, M	>50 nm
(T) Trapping	yes, particles at halt	tracking via side camera	C, R, M, ICP-TOF-MS^17^	>200 nm
(R) Raman	yes, velocity decrease	Raman signal via side camera	F, C, T, M	>250 nm
(B) Brownian	weak	tracking via side camera	C, M, analyze fluctuations	>50 nm
(M) Multiparticle SLS	no	SLS intensity via side camera	F, C, T, R, B	<50 nm
(O) Obscuration	no	tracking via front camera	extract shape and size	>1 μm

a We indicate the role of optical forces, how the OF2i signal is obtained, and provide an estimate for the particle sizes susceptible to the respective modes for refractive indices *n*_*p*_ in the range between 1.4 and 1.8. For particles with larger refractive indices the estimated sizes can be smaller.

The *sorting or trapping mode* is a variant of the previously discussed flow operation mode, where the direction of the fluid flow is reversed with respect to the laser propagation direction.^17^ As can be inferred from eq 5, for opposite orientations of ***v***_fluid_ and ***F***_tot_, the particles are slowed down with respect to the fluid velocity and can even come to a complete halt at size-dependent propagation or retention distances where the optical and fluidic forces have equal strength but opposite orientations. This approach has previously been referred to as optical chromatography,^48,49^ as it allows one to spatially separate and sort particles at different size-dependent positions.

Slowing down or sorting of particles plays an important role in the *Raman mode* for measuring Raman spectra, where longer measurement times are needed to obtain spectra with sufficiently high signal-to-noise ratios. In the top of Figure 4, we show the side image for three PS spheres in the trapping mode, which are brought to a halt at different positions depending on the respective sizes. The lower part of the panel reports the Raman spectra acquired.^17^ In addition to the broad background signal associated with water, which extends over the entire measurement cell, we observe at the positions of the particles Rayleigh and Raman signals, as shown in a video in the Supporting Information. The latter are enlarged in panel (b) and are representative of the chemical composition of the particles. Comparison with a database for Raman spectra allows us to assign the spectra to a polystyrene reference. Figure 4(c) shows results for TiO_2_ nanospheres,^17^ where we can assign the Raman spectra to those of the anastase material phase. Thus, from the trapping position and the Raman spectra, we can infer both the particle size and material composition as outlined in panel (d) of Figure 4.

**Figure 4:**
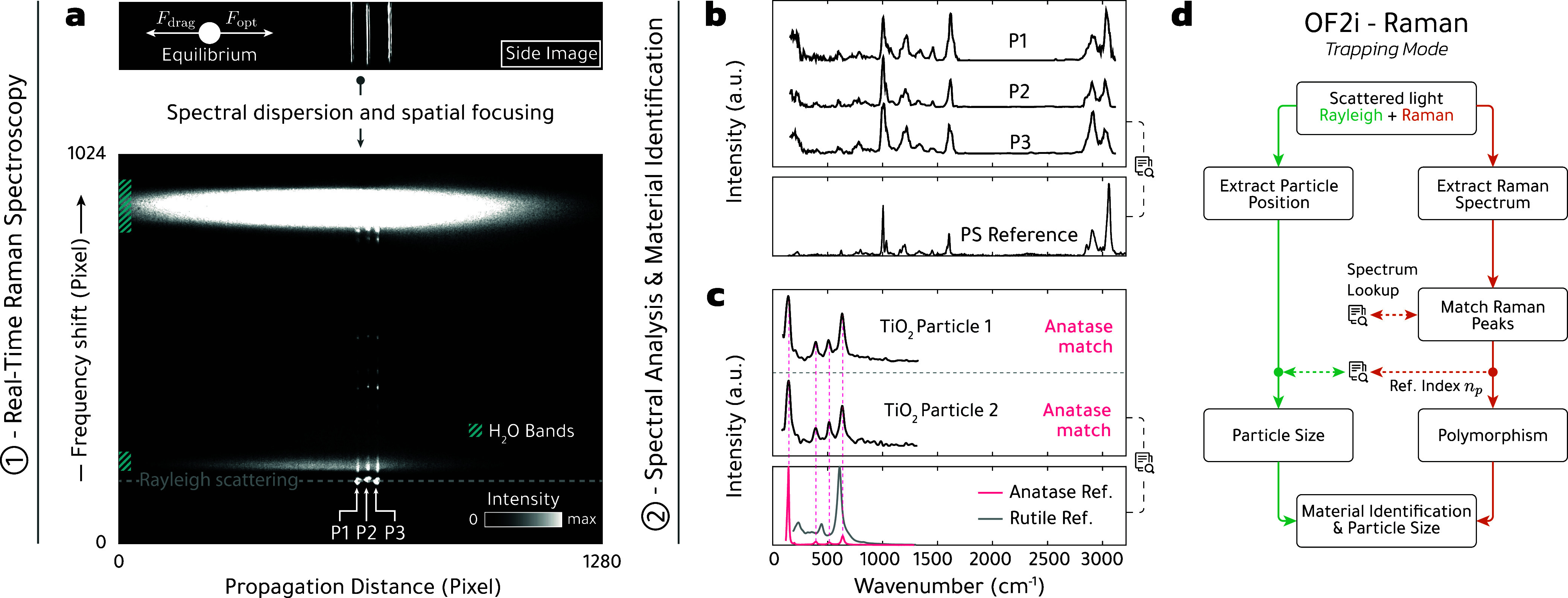
Real-time Raman spectroscopy and data processing (1), OF2i-Raman results, analysis, and material identification process (2). (a) Rayleigh scattering signal of three individual PS particles (P1–P3) trapped at size-dependent positions along the flow cell. The light scattered off particles is dispersed through a custom-built spectrometer, focused, and recorded using an imaging camera. A recorded image is shown as an example in the bottom panel. This image encodes the frequency shift (Stokes scattering) as a function of particle position. Note the Rayleigh scattering (dashed line) and the signals stemming from the water bonds (blue hatched areas). By extracting the intensity along the vertical axis, one obtains for each particle a Raman spectrum as shown in (b). We compare each spectrum against a lookup table that allows for direct material identification as PS. (c) Raman measurement of two TiO_2_ particles identified as the anatase polymorph. Adapted from ref 17. Copyright American Chemical Society. (d) Flow diagram illustrating the workflow and data processing stages for the extraction of particle characteristics within OF2i-Raman in trapping mode.

In ref 17 we have demonstrated that the trapped particles can be additionally released and transported into a mass spectrometer in order to obtain the elemental composition in addition to the previously determined size and Raman information. To this end, the water in the flow cell must first be replaced by ultrapure water, which is done while keeping the particles trapped, before releasing them to an inductively coupled plasma-time-of-flight-mass spectrometer (ICP-TOF-MS). Such complementary analysis promotes comprehensive particle characterizations with the most detailed elemental information about the analytes.

In the *correlative mode*, one records in addition to the particle velocity also the scattered light intensity. Similarly to iNTA,^32^ this approach provides information about the size of the particle and the refractive contrast. Figure 5(a) shows the measured scattering intensity versus particle velocity for particles in the focus region and for the oil-in-water emulsion shown in Figure 3 with its broad PSD. The solid line reports the corresponding simulation results that perfectly match the experimental data. The intensity-to-velocity curve depends sensitively to small changes of the refractive index of the scatterer, and thus allows determining material properties from the OF2i measurements without any prior knowledge.

**Figure 5:**
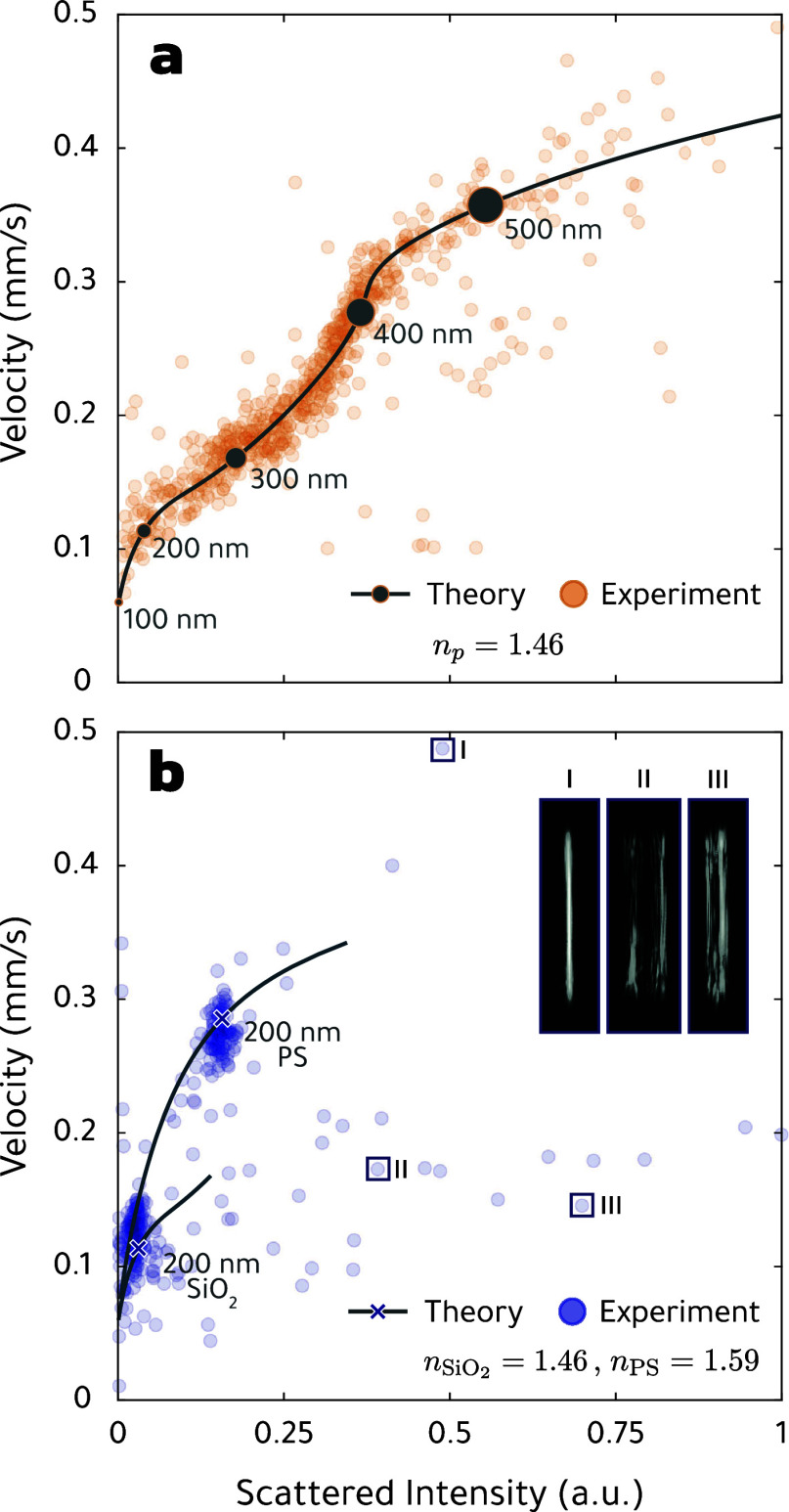
Continuous monitoring of polydisperse and multimodal samples by correlative OF2i. (a) Relation between particle velocity *v*_foc_ and scattered light intensity of an oil-in-water emulsion (*n*_*p*_ = 1.46). We compare experimental data with theory (solid line)^44,45^ across a broad range of particle sizes. Selected size classes of the simulation results are marked by gray dots, which scale with the corresponding diameter. (b) Measurement results for a mixture of silica (*n*_*p*_ = 1.46) and PS (*n*_*p*_ = 1.59) particles with nominal diameters of 200 nm. The scatter plot shows a distinct separation in particle velocity and scattered light intensity, supported by simulations (solid lines). Theoretical values corresponding to the nominal diameters are marked by a cross. The insets show side images corresponding to three selected outliers (I–III), which exhibit characteristic scattering patterns. In the experiments and simulations we use a focused Gauss beam.

Figure 5(b) shows correlative OF2i results for PS and SiO_2_ standard particles with well-defined sizes, which are again perfectly matched by our simulation results. In our measurements, we additionally observe several data points that are far off the theoretical curve and are attributed to particles composed of different materials or particle agglomerates. Since in OF2i each particle is observed while propagating through the microfluidic channel, we can go back to the raw videos recorded as side images and analyze the line shapes in more detail. Particle I in the inset of Figure 5(b) has a line shape similar to those of the side image in Figure 3(a), and is attributed to a spherical particle with a larger refractive index than polystyrene. In contrast, particles II and III exhibit line shapes that are broadened or structured, and are attributed to intrinsic *pollutants* in the fluid, such as particle clusters or agglomorates.

We close this section with a brief discussion of three additional operation modes that are currently under exploration. In our discussion so far, we have ignored Brownian motion, although it is important for small particles and is at the heart of other nanoparticle characterization schemes such as DLS or NTA. We have shown theoretically^44^ that Brownian motion does not impact the performance of OF2i for sufficiently large particles. For small particles, Brownian motion leads to an on- and off-hopping of particles to the intensity maxima of the focused laser beam, which translates to intensity fluctuations. These signals in the *Brownian mode* provide a unique fingerprint about the nanoparticle size and could be used for characterizing small particles that do not experience significant velocity enhancements, similar to the approach presented in ref 50. Preliminary results indicate that the intensity fluctuations are strongly enhanced for nonspherical particles because of rotational Brownian motion and could be used for extracting geometry information from the analysis of light scattering fluctuations.

The *multiparticle* *SLS* *mode*, which can be combined with most of the other OF2i operation modes, analyzes the background signal recorded in the side image, and provides information about small particle scatterers that cannot be discriminated individually. Preliminary results for samples containing small proteins (*d* ≈ 5 nm) show that the background scattering signal scales with size and concentration. For a given concentration, sizing and aggregation information is therefore obtainable.

Finally, in the *obscuration mode*, the focused laser is replaced by the incoherent illumination of a light-emitting diode that excites the entire capillary, and the particles pumped through the microfluidic channel are imaged using a camera in the forward direction, see Figure 2(a). Sufficiently large particles, say with diameters above 1 μm, can then be detected through their obscuration in the focus plane of the camera objective. As shown in a video in the Supporting Information, one can use an image processing algorithm to count each particle while it is transported along the flow cell, and obtain with high accuracy the particle concentration, size distribution, and shape information.

## Summary and Outlook

To summarize, we have presented OF2i as a versatile platform technology that provides deep insights into nanoparticle characteristics and can be used with a wide range of operation modes. With the ability to analyze monodisperse, polydisperse, and multimodal samples with high precision and adaptability, it serves as a valuable tool for basic research as well as industrial applications.

OF2i comes along with a number of limitations that are either inherent to the technique or need to be overcome in future work. First, because of the monitoring of individual particles in real time, the maximum number of tracked particles per minute should not exceed 1000, corresponding to particle concentrations of less than about <10^10^ particles/mL. For samples with higher concentrations, dilution is required, a process that introduces a time lag of about two to 3 min in the monitoring process, depending on the required dilution ratio and sample availability. Second, the extraction of particle sizes using optical forces restricts the applicable size range depending on the refractive index of the sample under investigation. The lower limit is determined by too small scattering forces, which scale with the size *d* as *F*_scat_ ∝ *d* ^6^, and the upper limit is currently dictated by Mie resonances, which lead to a nonmonotonic relation between particle size and velocity enhancement. The previously described correlative measurement approach of velocity enhancement and scattering intensity might allow one to partially overcome these limitations in the future.

Finally, in the past we have predominantly used OF2i for the analysis of dielectric nanoparticles with homogeneous material properties, low or no absorption, and sharp interfaces. For absorbing particles, we expect heating effects to occur, resulting in photophoretic forces that depend sensitively on the particle size, which are currently not considered in our theoretical framework. Additionally, for metallic particles, we find that the gradient forces do not provide a stable trapping in the transverse directions, a finding in agreement with optical tweezer experiments. Future work will also address the influence of optical forces on core–shell nanoparticles, which are of high interest in nanomedicine applications, determining loading states for the development of targeted drug delivery systems using liposomes, virus-like particles, and particle synthesis processes where OF2i allows the dynamic detection of various particle populations and growth rates over large time scales.

Capable of real-time particle size distribution measurement, concentration determination, shape characterization, and material identification over a broad measuring range, OF2i offers a comprehensive solution by combining high accuracy and flexibility across different sample types. It has potential as a convenient and effective analytical method for detecting high-priority contaminants such as nano- and microplastics. Its role in enhancing process analytical technology and ensuring regulatory compliance further underscores its impact in developing safer, higher-performing products in pharmaceuticals, biotechnology, industrial manufacturing, and semiconductor applications, contributing to a safer and more sustainable future in nanotechnology-driven industries.

## Supplementary Material









## Notes

OF2i obscuration operation mode (MP4)
OF2i flow operation mode (MP4)
OF2i-Raman operation mode (MP4)
Brief description of supporting video material showcasing OF2i operation modes (PDF)
