# Supplementary material for: Optofluidic Force Induction: A Workbench for Nanoparticle Characterization and Material Analytics
Source: Nano Lett. 2025 May 21;25(22):8805–13. doi: 10.1021/acs.nanolett.5c01126 (PMC12142671; doi:10.1021/acs.nanolett.5c01126)
Supplement: Supplementary file 4 [file nl5c01126_si_004.pdf]

Optofluidic force induction: A workbench for nanoparticle characterization and material analytics: Supplementary Information

Marko Šimić,[1,2,*] Christian Neuper,[3,4] Raphael Hauer,[1,3]
Karin Grießmair,[3,5] Christian Hill,[3,6] and Ulrich Hohenester[1]

[1] *Institute of Physics, University of Graz, Universitätsplatz 5, 8010 Graz, Austria*

[2] *Christian Doppler Laboratory for Structured Matter Based Sensing, Institute of Physics, Universitätsplatz 5, 8010 Graz, Austria*

[3] *Brave Analytics GmbH, Austria*

[4] *Graz Centre for Electron Microscopy, Steyrergasse 17, 8010 Graz, Austria*

[5] *Institute of Biomedical Imaging, Graz University of Technology, Stremayrgasse 16/III, 8010 Graz, Austria*

[6] *Gottfried Schatz Research Center, Division of Biophysics, Medical University of Graz, Neue Stiftingtalstraße 2, 8010 Graz, Austria*

In this Supplementary Information, we briefly describe the video material showcasing various OF2i operation modes, provided as supplementary material to the main text.

3 pages S1–S3

0 figures

0 tables

* marko.simic@uni-graz.at

Video Description: OF2iPolyDemo.mp4

In this video, we show an excerpt from the recorded scattered light intensity from the side image, obtained by continuously monitoring a multimodal and polydisperse mixture of polystyrene particles (see also Fig. 3a–e in the main text). Note that in the video the particle flow and the laser propagation direction is from right to left, and thus flipped in comparison to Figs. 2–4 of the main text. The scattered light of individual particles is imaged as lines due to the cylindrical shape of the capillary. Larger particles, which appear brighter in the video, move faster within the focal region of the laser beam (approximately at the center of the image). Due to the vortex beam used in our experiments, particles can overtake each other, while collisions are significantly suppressed.

Video Description: OF2iRamanDemo.mp4

This video demonstrates the working principle of the OF2i Raman mode. Light scattered in the side direction is first spectrally dispersed and then recorded using a scientific CMOS camera, see also Fig. 4 of main text. The left panel shows the real-time evolution of the recorded Raman signal. The horizontal axis provides information about the axial position of the particles within the capillary, while the vertical axis corresponds to the Raman shift. Notably, due to the constant presence of water, its characteristic Raman signal is detected throughout a measurement. Once a particle enters the field of view, we track it via its Rayleigh signal and extract the corresponding Raman shifts over time. Finally, the temporal mean of all spectra for a single particle is computed and displayed in the right panel.

Video Description: OF2iObscurationDemo.mp4

This video demonstrates the working principle of the OF2i obscuration mode. We use an incoherent light source for illumination and record light exiting the capillary through its rear laser window using a converging lens and a camera. Due to the continuous sample flow, particles larger than 1 μm eventually create a sharp obscuration as they move towards the focus plane of the imaging system. The video shows raw data annotated by an in-house developed detection and sizing scheme, which allows for real-time determination of particle concentration (see right panel) and particle size distribution (not shown). Particle sizing

is achieved by measuring the area of the obscured patch at maximum contrast. For this demonstration, a NIST-traceable polystyrene particle standard ($n_p = 1.59$) with a nominal diameter of 5 μm and a concentration of 3000 particles/mL was used.
